# A Digital Approach for a Complete Rehabilitation with Fixed and Removable Prostheses: A Technical Procedure

**DOI:** 10.3390/dj13010007

**Published:** 2024-12-25

**Authors:** Etienne Lefrançois, Victor Delanoue, Samuel Morice, Xavier Ravalec, Marie Desclos-Theveniau

**Affiliations:** 1 Department of Prosthodontics, University of Rennes, 35043 Rennes, France; 2 University Hospital, CHU de Rennes, 35033 Rennes, France; 3 UMR 6226, CNRS, Rennes Institute of Chemical Sciences, University of Rennes, 35700 Rennes, France; 4 Private Practice, 35051 Cesson-Sevigné, France; 5 Private Dental Laboratory ARGOAT, 22970 Ploumagoar, France; 6 U1317 INSERM, Institut National de la Santé et de la Recherche Médicale, 35033 Rennes, France

**Keywords:** digital dentistry, fixed partial denture, removable partial denture, CAD/CAM frameworks

## Abstract

**Background:** The present article describes a step-by-step maximally digitalized workflow protocol with computer-aided design and computer-aided manufacturing (CAD/CAM) in partial-arch edentulous patients rehabilitated with fixed dental prostheses and removable partial dentures (FDPs and RPDs). **Methods:** Facial digitalization, intraoral scans, and functional mandibular movement recordings were used to create a 4D virtual patient on commercially available CAD software. The fixed components including post-and-cores, both metal–ceramic with extra-coronal attachment and monolithic zirconia crowns, and the RPDs were manufactured by computer numerical controlled direct milling. **Results:** This innovative digital approach using the virtual patient and the superimposition of interim RPDs fitted in the mouth has been used to provide fixed and removable rehabilitation to the patient without clinical complications with 2 years of follow-up. **Conclusions:** Within the limitations of this report, the developed combined prosthesis fabrication technique allowed optimization of the production by decreasing the clinical steps and laboratory procedures in partial-arch edentulous rehabilitated with FDPs and RPDs.

## 1. Introduction

Removable partial dentures (RPDs) combined with fixed dental prostheses (FDPs) remain as one of the more conservative and cost-effective treatments for many partially edentulous patients [1,2]. With the conventional method, this option is time-consuming and stressful for the practitioner. It also involves complex laboratory procedures unchanged for many decades despite the clinical and laboratory errors that can be accumulated during the procedure [3,4,5].

Nowadays, the use of computer-aided design and computer-aided manufacturing (CAD/CAM) is more established in FDP protocol than in the RPD process [6,7]. The incorporation of digital technologies in partial denture is on the rise both by dentists with intraoral scanners (IOSs) and by dental technicians with the design and fabrication of RPD components on three-dimensional casts [8,9,10,11,12,13,14,15,16,17]. Recently, two systematic reviews have summarized the digital workflow of RPD using evidence from clinical trials and case reports [8,9]. However, studies on the implementation of a fully digital workflow in partial-arch edentulous patients rehabilitated with both FDPs and RPDs are scarce.

Fully digital workflow studies are based on a virtual patient created by a superimposing facial scan, digital dental arch impressions, maxillomandibular relationship (MMR), and more recently, functional mandibular movements with the jaw motion tracer [18,19,20,21,22]. At the current moment, the digital impression technique does not allow the recording of soft tissue pressure displacement on removable denture-bearing areas [8,23]. Conventionally, a selective pressure impression on the residual ridge is required to fabricate well-fitted RPD with extension base(s) for Kennedy class I and II arches. Previous articles have described altered cast impression methods based on a stone definitive cast, prefabricated teeth, and a physical articulator [24,25,26,27].

This report describes the use of a maximally digitalized workflow from the planning stage to the final fit in partial-arch edentulous patients rehabilitated with FDPs and RPDs. It also develops a method of acquiring the functional outline of the denture base.

## 2. Materials and Methods

The technical procedure is illustrated by a 50-year-old woman referred to the Department of Prosthodontics for prosthetic rehabilitation. Her main expectation was complete functional rehabilitation with fewer demands concerning esthetics. The patient did not have any systemic disease.

### 2.1. Initial Consultation

**Figure 1:**
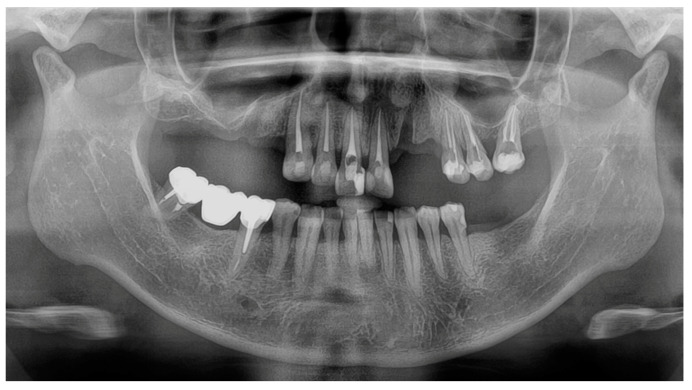
Initial panoramic radiography.

Clinical investigations showed partially edentulous regions of the maxilla (Kennedy class II, #14-17, #22-23, #27) and mandible (Kennedy class II, #36-37). Tooth #13 had a significant vestibular cervical lesion and the maxillary teeth had several composite restorations. Tooth #12 had a mesio-palatal rotation and tooth #44 had a slight extrusion. In the mandible, a satisfactory metal bridge replaced tooth #46. No alteration of the vertical dimension of occlusion (OVD) was observed. The smile line only revealed gingival embrasures (Class III according to Liébart classification). The patient did not wish to correct the misaligned gingival margins of the maxillary central incisors. The panoramic radiograph revealed that all the maxillary residual teeth had been treated endodontically, and teeth #15 and #22 had recently been removed (Figure 1). Tooth #27 was mesially positioned in place of tooth #26 probably due to an uncompensated extraction in the past.

**Figure 2:**
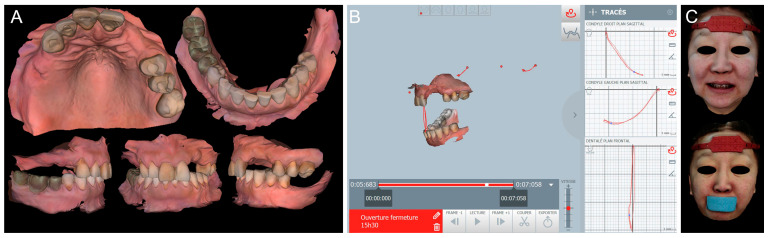
Digital view of the initial situation: (**A**) 3D images (TRIOS 4; 3Shape A/S) of maxillary, mandibular, and MMR captured by IOSs; (**B**) functional mandibular movements recording with the jaw motion tracer (MODJAW; MODJAW); and (**C**) facial scans (Bellus3D Face Camera Pro; Bellus3D) with AFT aligners.

Digital impressions of the patient’s existing maxillary and mandibular arches and the MMR were obtained using the intraoral scanner (TRIOS 4; 3Shape A/S) (Figure 2A). The digital complete-arch casts were imported and aligned into the jaw motion system (MODJAW; MODJAW). The mandibular movements were registered following the manufacturer’s protocol (Figure 2B). Finally, the patient’s face was digitized with the facial scanner (Bellus3D Face Camera Pro; Bellus3D) and the AFT (AFT dental system) protocol (face and teeth aligners) (Figure 2C) [19].

### 2.2. Creation of Virtual Patient and Treatment Plan

**Figure 3:**
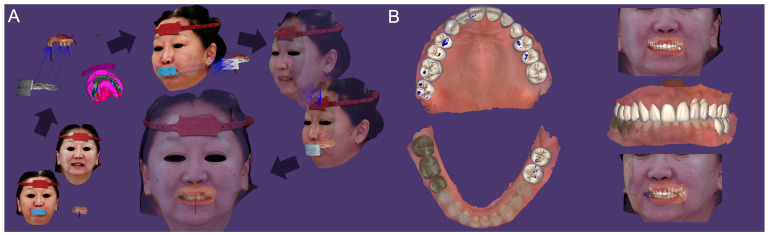
(**A**) Creation of a 4D virtual patient by aligning digital data on CAD software (Exocad DentalCAD; exocad GmbH) and (**B**) occlusal and frontal views of digital waxing.

A 4D virtual patient was created by importing the scan data and mandibular motion files into the CAD software program Version 2.3 (Exocad DentalCAD; exocad GmbH). Data were aligned for the rehabilitation of the maxillary and mandibular arches (Figure 3A). A diagnostic waxing of the residual teeth and artificial teeth on the edentulous ridges was designed according to esthetic and functional parameters (Figure 3B). The static and dynamic occlusions were then adjusted and validated with the digital articulator and the mandibular motion.

In this case, the restorative space was sufficient without increasing the OVD to obtain a correct prosthetic rehabilitation. Casts of the virtual wax-up were printed to communicate with the patient and then to produce mock-ups and preparation indexes. The patient was informed of available treatments including maxillary teeth rehabilitated with crowns, and missing teeth replaced with implants or a removable partial denture. She expressed a fear of implant surgery and financial restrictions.

With the patient’s consent obtained, a treatment plan including maxilla and mandibular RPDs and fixed dentures for all maxillary teeth was implemented. Porcelain fused to ceramic FDPs from tooth #13 to #21 was chosen with an extra-coronal attachment on tooth #13 in order to avoid the unsightly clasp in this area and to provide effective retention of the RPD. Joined monolithic zirconia crowns were selected to restore teeth #24 to #26.

### 2.3. Fabrication of Interim RPDs and FDPs’ Restorations

Once the treatment plan has been approved, the interim rehabilitation was created by the dental technician on the virtual patient. A 5-axis computer numerical control (CNC) dental milling machine (CNC 308B; Willemin-Macodel) was used to mill the RPD teeth (personalized teeth based on the dynamic records) and shells of temporary FDPs based on the design of waxing using a tooth-shade PMMA block (Aidite Temp PMMA multilayer; Aidite). The interim RPDs were finished conventionally (clasps and denture base) with the lost wax technique.

### 2.4. Preparation of Teeth and Interim Restorations

**Figure 4:**
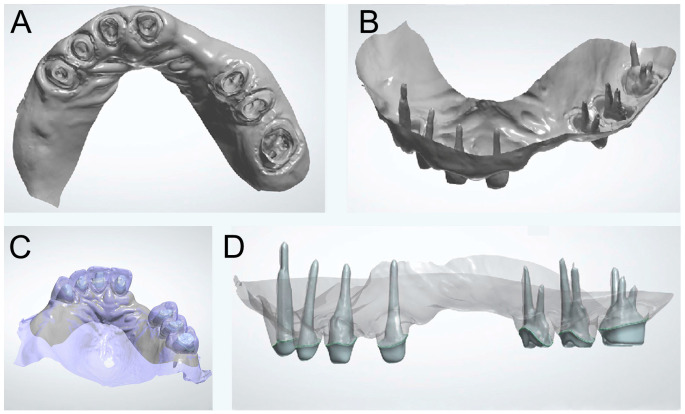
Digital impression of the entire root preparations without scan posts (Primescan; Dentsply Sirona): (**A**) external and (**B**) internal digital maxillary view of root canal preparations; (**C**) digital impression merged with digital waxing; and (**D**) post-and-cores digital design (3Shape Dental System; 3Shape).

A mock-up was applied to the maxillary arch with a bisacryl temporary material. To obtain an optimal tooth reduction according to the material of choice (metal–ceramic crowns for the anterior teeth and monolithic zirconia for the posterior teeth), the maxillary teeth were prepared through the mock-up and checked using preparation indexes from the wax-up. The root preparations were made considering the amount of residual tissues. A coronoplasty of tooth #44 was also made according to the wax-up in order to restore a harmonious occlusal curve. A digital impression of prepared teeth using an IOS (Primescan; Dentsply Sirona) without scan posts was obtained (Figure 4A,B). Temporary shells were relined and fitted onto the dental abutments (adding posts if necessary for retention of the device). Stability, retention, esthetic, phonetics, and occlusion were evaluated after FDP cementation and RPD insertion.

### 2.5. Post-And-Cores

Digital data from the last step were imported into the CAD software. They were then superimposed with the virtual diagnostic waxing by using the iterative closest point technique (Figure 4C). The right and left medial points of the third rugae and one point in the middle of the incisive papilla were the three landmarks selected as common points in both files. Anatomic post-and-cores were designed (Figure 4D) and manufactured with a 5-axis CNC dental milling machine (CNC 308B; Willemin-Macodel) and a CAD/CAM titanium disc (Nicrallium TA6V Grade 23; BCS Dental Alloys). The parts have been fully anodized and sandblasted in the intra-canal part with 150 µm aluminum oxide particles. The post-and-cores were tried-in intraorally and cemented (Fujicem 2; GC). The temporary FDPs were re-adjusted using acrylic resin (Unifast; GC). The intaglio surface of both mandibular and maxillary RPDs was relined with an impression material (F.I.T.T; Kerr) to make the final impressions and to record the edentulous ridges more accurately. A follow-up of at least three weeks was required to evaluate the patient’s comfort and esthetic. Once the temporary restorations are validated, the definitive RPDs and FDPs can be achieved.

### 2.6. Optical Impressions and Merging

**Figure 5:**
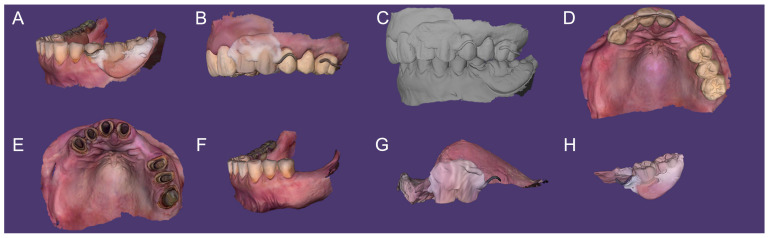
Digital impressions (TRIOS 4; 3Shape A/S): (**A**) mandibular arch with prosthesis (relined RPD); (**B**) maxillary arch with prostheses (FDPs and RPDs relined); (**C**) maxillomandibular relationship recording (with all dentures); (**D**) maxillary arch with FDPs; (**E**) maxillary arch without prosthesis; (**F**) mandibular arch without prosthesis; (**G**) maxillary RPD scanning out-of-mouth; and (**H**) mandibular RPD scanning out-of-mouth.

Intraoral scans (TRIOS 4; 3Shape A/S) of mandibular arch with the relined RPD (Scan A), maxillary arch with FDPs and relined RPD (Scan B), MMR record with prostheses (Scan C), maxillary arch with FDPs (Scan D), maxillary arch without prostheses (Scan E), and mandibular arch without RPD (Scan F) were obtained (Figure 5). Relined maxillary (Scan G) and mandibular (Scan H) RPDs were also scanned extra-orally (Figure 5). And the dynamic occlusion and tooth color were registered.

**Figure 6:**
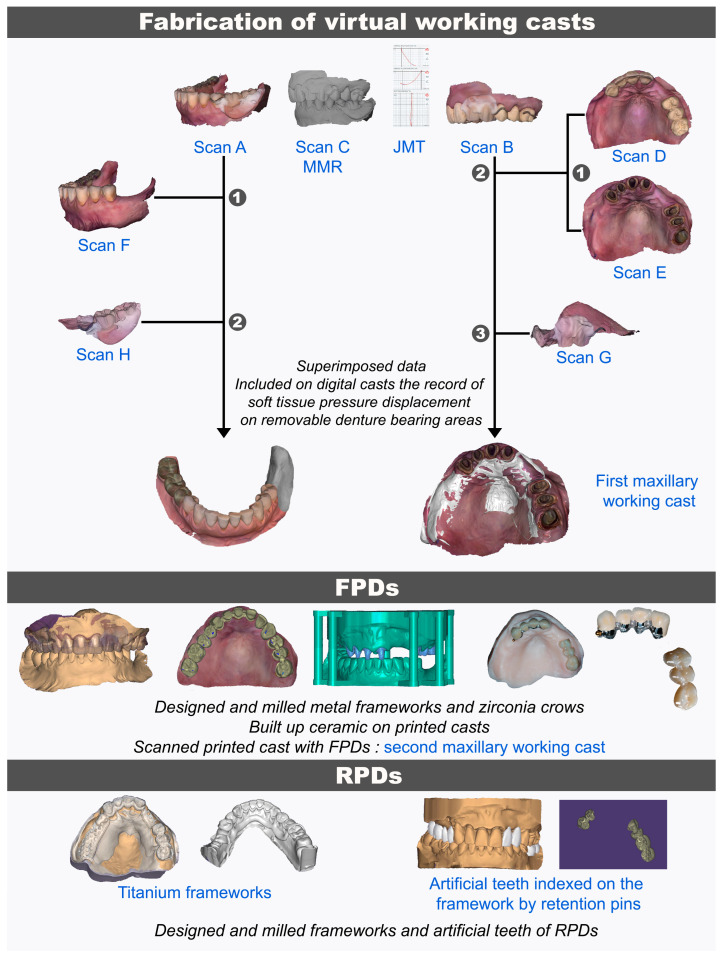
Digital workflow based on a 4D virtual patient in a partial-arch edentulous patient rehabilitated with FDPs and RPDs. Numbers: chronology of matching. MMR: maxillomandibular relationship; JMT: jaw motion tracer.

The aforementioned digital data were uploaded into the CAD software. For the maxillary arch rehabilitation, Scans D and E were merged using palatal landmarks previously described, then superimposed with Scan B and Scan G to create virtual working casts (Figure 6). The same protocol was performed for the mandibular arch. If needed, the scan position was further adjusted manually. The edentulous ridge surfaces were erased on the virtual casts, and the denture-bearing areas were substituted with the relined intaglio of the RPDs (Scans G and H). The working cast thus includes an accurate record of soft tissue displacement and border molding (Figure 6).

### 2.7. Fabrication of Definitive FDPs

Metal–ceramic crowns and the monolithic zirconia crowns were designed from the working cast. For metal–ceramic crowns, the framework was designed and milled in Cobalt Chromium discs (BionMet CCW; Bionah Srl) including a screw thread distally in order to add a VK screw system as an extra-coronal attachment on tooth #13 (Figure 6). The metal framework was inserted onto a printed working cast to build up the ceramic on teeth #13 to #21, assisted by a silicone index derived from the wax-up. Joined monolithic zirconia crowns for teeth #24 to #26 were also cosmetically enhanced through external staining and glazing of the surfaces. The extra-coronal attachment (Vario-Kugel-Snap vks-sg exchangeable stud; Bredent) was screwed into the FDP framework according to the manufacturer’s instructions. FDPs were tried-in intraorally to validate esthetic, phonation, and fit. The FDP components were placed on the printed cast (Figure 6) and scanned (E3 lab scanner; 3Shape A/S) to obtain a second working cast for the RPDs.

### 2.8. Fabrication of Definitive RPDs

The RPD frameworks and the artificial teeth (customized and indexed on the framework by retention pins) were designed from the second cast (Figure 6). The artificial teeth (Aidite PMMA multilayer; Aidite) and titanium framework (Nicrallium TA6V Grade 23; BCS Dental Alloys) were milled. The teeth were then manually bonded with resin (Probase hot; Ivoclar Vivadent) conventionally with a lost wax technique.

**Figure 7:**
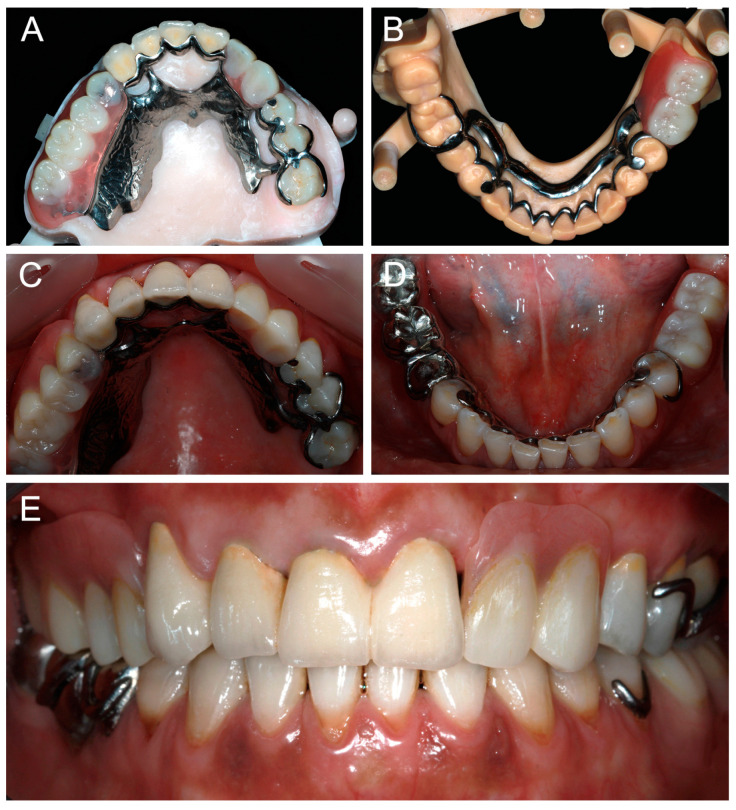
Occlusal views of maxillary and mandibular definitive prostheses: (**A**,**B**) on the printed casts; (**C**,**D**) in clinical situation; and (**E**) frontal view of prostheses in clinical situation.

FDPs and RPDs were tried-in intraorally. Stability, retention, esthetic, phonetics, and occlusion were evaluated. Minor adjustments were made where needed. FDPs were definitively cemented and the RPDs were inserted (Figure 7). Occlusion was again verified. Maintenance instructions and oral hygiene methods were provided to the patient.

**Figure 8:**
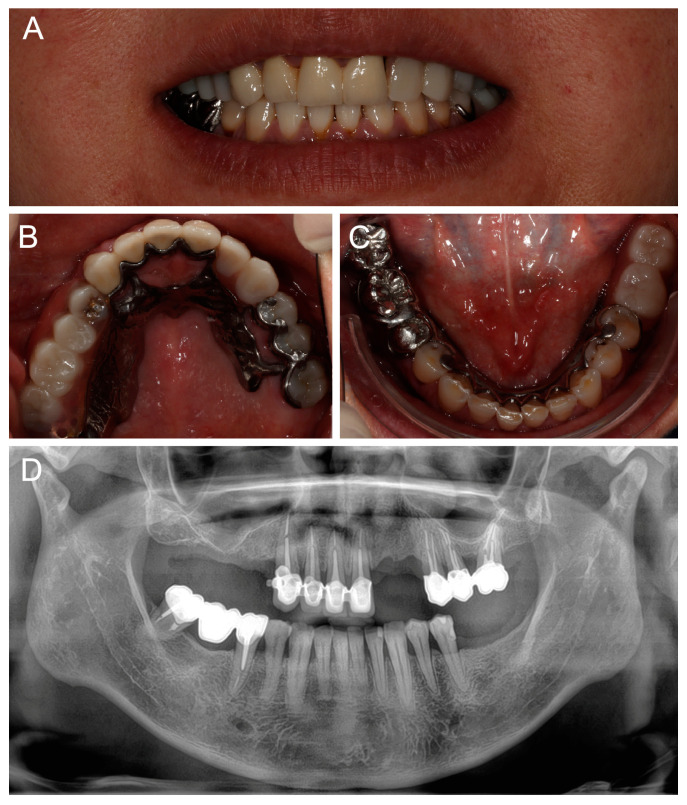
Follow-up at 2 years: (**A**) smile view; (**B**,**C**) occlusal views of maxillary and mandibular definitive prostheses; and (**D**) panoramic radiography.

At a 2-year follow-up, the patient was completely satisfied with the rehabilitation and expressed no complaints (Figure 8). The maxillary RPD clasps were slightly retightened on teeth #25 and #26, and standard periodontal maintenance was carried out.

## 3. Discussion

### 3.1. Difference from Conventional Methods

The maximally digitalized workflow protocol for patients rehabilitated with both RPD and FDP allows advantages over the conventional method. This procedure simplifies the process, decreases inaccuracy caused by complex laboratory procedures unchanged for many decades, reduces clinical steps, and improves communication between clinicians and technicians [8]. This article describes the minimum number of appointments. If the FDP and RPD fittings are not validated at the first try-in session, additional steps could be required. However, this technology makes procedures quicker with or without modifications compared to conventional methods. In this clinical situation, three fewer appointments were required (representing about a 20% reduction in the number of appointments) for the patient compared to the conventional method. This reduction is primarily due to the elimination of multiple MMR records required at different stages of these complete rehabilitations. The cost of the treatment remains the same for the patient as with the conventional method. For the practitioner, preserving the initial MMR record throughout the treatment (by matching digital impressions) saves time and reduces the risk of errors by minimizing the number of steps. At the laboratory level, the simultaneous fabrication of multiple prostheses simplifies the management of such procedures (e.g., color harmony, and the adaptation of the different prostheses to one another). As a result, the overall working time is reduced, with CAD/CAM processes providing a significant reduction in time compared to traditional methods, with an estimated time savings of more than 50%.

To fabricate well-fitted Kennedy class I and II RPD, border modeling is essential to record soft tissue functional movements and pressure displacement of mucosa on denture-bearing areas [8]. Current IOSs cannot capture correctly soft tissues, especially in a functional state. A modification of an existing method based on an altered cast impression is used in the present technique to determine the outline of the denture base [27]. Relined RPD is integrated with intraoral scans of dentition and mucosa to create a definitive digital cast. In this digital procedure, interim or existing RPDs with well-fitting were necessary for fabricating the new RPDs. Without inadequate extensions of the edentulous areas, this technique will not be successful. The fitting accuracy for the definitive RPDs depended on the protocol of intaglio surface relining and the superimposition process on CAD software. Prosthesis relining allowed to capture correctly adequate extensions of the edentulous areas and intaglio surface. If the patient does not wear any prosthesis, additional steps are required to obtain a precise impression of soft tissue using an individual impression tray. Moreover, digital alignment needs practice and familiarity with the technical platform. It can be complicated and time-consuming for the novice. These clinical and technical challenges can be considered as limitations for this technique.

No scan post was used to make impressions in this clinical situation. The Primescan (Dentsply Sirona) used is the most efficient IOS that can currently obtain accurate impressions of root preparations without using scan posts. An in vitro study has demonstrated that this IOS was able to record post-preparations with a depth lower than 14 mm with a diameter of over 2.2 mm, simplifying the impression procedures without altering the fit compared with a conventional method [28]. Other modern IOSs are suitable for recording intra-canal preparations for post-and-core, such as the TRIOS 4 [29], but can be trickier to use in some clinical situations. With root preparations less than 11 mm in depth, it was easier to use the Primescan for this step.

The complex jaw relation-transferring conventional procedure based on the facebow technique, additional registration materials, and mechanical articulators is a step neglected by some clinicians. IOSs do not allow complete registration of the dynamic occlusion, and consequently, a lack of an adequate occlusal concept could cause prosthetic instability. This digital workflow integrates the jaw motion tracer to record and preserve the dynamic occlusion throughout the treatment. This registration leads to a more precise design of artificial teeth on RPDs and FDPs with occlusal anatomy during functional mandibular movements and, consequently, reduces direct occlusion adjustment by chairside [30]. The harmonious occlusal relationship is recommended to enhance the stability of the removable partial dentures. In this clinical situation, the occlusion recorded from the outset was maintained throughout the treatment and reinforced by the rehabilitation. No record base was necessary to record the MMR. The occlusion of convenience corresponded to the occlusion of centric relation. Minor adjustments in the occlusion were necessary during the delivery of prostheses.

In this present method, the direct milling framework by computer numerical control eliminates possible errors of traditional casting and offers at least a good fit accuracy compared to conventional techniques [9]. No adjustments were required for the insertion of the RPDs, which were correctly adjusted with both the crowns and the soft tissue. Titanium alloy also brings an incomparable lightness to the framework. It has been shown that the lower elastic modulus of titanium alloy rather than cobalt–chromium leads to better root stress distribution, especially for post-and-core restorations [31,32]. However, some authors have reservations about the precision of milled clasps for RPD [33].

### 3.2. Effect or Performance

The most important feature of this method is that the digital workflow procedure used for complete arch rehabilitation in patients with fixed and removable prostheses simplifies both the design and production of the prosthesis process. The use of the virtual patient enabled efficient communication with the patient and an informed decision to be taken on an overall treatment plan. In this situation, the patient did not want the asymmetry of the maxillary incisor gingival margins to be corrected and was able to visualize this on the virtual patient. For this rehabilitation, the limited amount of residual tissue and the use of RPD retained with an extra-coronal attachment have required the use of crowns and post-and-cores. Whenever possible, therapeutic options with the most conservative approach should be preferred at present.

CAD/CAM is useful to minimize the number of appointments, the dental lab working time, and the materials used [8]. Intraoral scans, facial scans, jaw motion tracking systems, and virtual articulators are known to be more efficient compared with the traditional workflow [8,9]. However, the superimposition of multiple digital impressions can lead to errors during the various matching processes [21]. Specific anatomical structures on the palatal surface are known to be reliable and stable for the superimposition of dental casts [34,35]. These structures are therefore used when there are no matching dental surfaces between the different impressions. Furthermore, the different files should be used for which they were generated. For example, the face scan is used to determine the ideal position of the midline, the position of the occlusal plane in the frontal and sagittal planes, and overall esthetics [36]. The design of restorations is preferably dictated by the occlusion recorded with the IOS and refined by the recording of mandibular movements. The matching protocol used in this case for the facial scan involves using a fork with dental support and extra-oral markers and then a laboratory scanner. Other protocols have been developed to facilitate this stage [36]. The alignment step can affect the virtual model’s accuracy [21]. In this clinical case, the challenge is to create the merged model of the teeth and the intrados of the RPDs. By taking all the different impressions in the protocol, it is possible to use the software’s different superimposition processes (by point, by surface, manually, etc.) to optimize each stage of merging. Simplifying the procedure of occlusion recording and transferring is essential during the complex rehabilitation procedure. The jaw motion tracer allows monitoring of the jaw positions and movements in real time or the determination of a new OVD within the range of hinge movement [37]. This is the first case report to use a maximally digitalized workflow for the fabrication of an RPD and FDP rehabilitation.

Among limitations, this digital treatment requires process skills and laboratory experience. The initial acquisition of digital devices remains expensive. Well-designed clinical trials are necessary to evaluate the efficacy and usefulness of digital RPDs. More studies are also required to determine the accuracy of the digital MMR in clinical routine.

## 4. Conclusions

An innovative digital approach using the virtual patient and the superimposition of interim RPDs fitted in the mouth has been used to provide fixed and removable rehabilitation to the patient without clinical complications with a 2-year follow-up. Almost all fixed and removable restorations were milled (post-and-core, framework incorporating a screwed extra-coronal attachment, zirconia crowns, and frameworks and teeth of RPD). Within the limitations of this report, the developed combined prosthesis fabrication technique allowed optimization of the production by decreasing the clinical steps and laboratory procedures in partial-arch edentulous.

## Data Availability

The original contributions presented in this study are included in the article; further inquiries can be directed to the corresponding author.
